# Absence of neurotoxicity with perineural injection of ultrasound gels: assessment using an animal model

**DOI:** 10.1186/1471-2253-13-18

**Published:** 2013-09-03

**Authors:** David Belavy, Nana Sunn, Queenie Lau, Thomas Robertson

**Affiliations:** 1 Burns, Trauma and Critical Care Research Centre, The University of Queensland, Royal Brisbane and Women’s Hospital, Butterfield Street, Herston, QLD 4029, Australia; 2 The University of Queensland Diamantina Institute, Translational Research Institute Pty Ltd, Princess Alexandra Hospital, Level 6, 37 Kent St, Woolloongabba, QLD 4102, Australia; 3 Department of Pathology, Royal Brisbane and Womens Hospital, Herston 4029, Australia

**Keywords:** Ultrasound gel, Neurotoxicity, Regional anesthesia, Ultrasound, Animal models, Nerve injury

## Abstract

**Background:**

Ultrasound gels may contain propylene glycol and glycerol, which are neurotoxic in high concentrations. If the needle passes through gel during regional anesthesia, gel may be injected near the nerve. It is unknown if this practice poses a risk for neurotoxicity. Using an animal model, we assessed the histological changes of perineural propylene glycol on nerves. We then assessed three commonly used sterile gels for evidence of neurotoxicity.

**Methods:**

Micro-ultrasound guided perineural sciatic nerve injections were performed in mice. Propylene glycol (PG) 2.5%, 10%, 35%, 70% (v/v) or saline was injected. Nerves were assessed after three days for evidence of neurotoxicity. Aquasonic® 100 Ultrasound Gel, K-Y® Lubricating Jelly, and PDI® Lubricating Jelly were also studied against saline controls.

**Results:**

Confluent areas of axonal degeneration and intraneural inflammation occurred in 5 of 9 specimens injected with 70% PG. At 35%, 2 of 8 specimens showed patchy changes not present at lower concentrations. No degeneration occurred with Aquasonic® 100 or PDI® Lubricating Jelly. In the K-Y® group, one gel and one saline specimen demonstrated confluent degenerative changes.

**Conclusions:**

Similar to glycerol, 70% PG may cause confluent areas of axon and myelin degeneration with associated intraneural inflammation. The concentration of PG present in ultrasound gels is unlikely to cause neurotoxicity. Aquasonic® 100 and PDI® Lubricating Jelly did not cause neurotoxicity. The results for K-Y® Lubricating Jelly are inconclusive. There is no evidence that passing the needle through the studied gels during regional anesthesia procedures is harmful.

## Background

Image quality in ultrasound guided regional anesthesia is improved by using ultrasound gel as a coupling medium between the probe and the patient’s skin. Some clinicians pass the needle through this sterile gel when performing nerve blocks. It has been demonstrated that regional anesthesia needles can carry macroscopic quantities of ultrasound gel into tissues [1]. As a result, ultrasound gel can potentially be injected around, or into, nerves.

The composition of ultrasound and lubricating gels is often unknown to clinicians but may include the viscous alcohols glycerol and propylene glycol (PG) [2-6].

Pure glycerol has neurolytic properties but the concentration present in ultrasound gels is probably below that associated with neurotoxicity [6-8]. Pure PG is also an effective neurolytic agent and has been proposed as an alternative to phenol [9]. In the 1950s, a long acting preparation of procaine, called Efocaine, was associated with tissue sloughing, cellulitis, lumbosacral neuritis and paraplegia [10-12]. The vehicle contained 78% propylene glycol and was demonstrated to cause nerve destruction in an animal model [13]. The lowest concentration of PG associated with neurotoxicity is unknown.

As the composition of ultrasound gels is often unknown, the potential for neurotoxicity with perineural injection is unknown. One ultrasound gel (Pharmaceutical Innovations, Newark, NJ) has been studied in a porcine peripheral nerve model and demonstrated no histological evidence of neurotoxicity [14]. Another study found that Eko gel (Eurocamina S.r.l., Salerno, Italy) injected intrathecally causes meningeal and spinal inflammation [15].

Other sterile gels in common use have not been evaluated. Similarly, the potential for neurotoxicity related to lower concentrations of propylene glycol has not been evaluated. This study assesses the concentration-response relationship between PG and histological nerve damage as well as the histological effect of perineural injection of three sterile gels on peripheral nerves.

## Methods

The study assessed the histological effects of perineural injections of propylene glycol and three medical gels in a mouse model utilizing ultrasound guided microinjection techniques.

The study was approved by the University of Queensland Animal Ethics Committee (Anatomical Biosciences) according to the principles of the National Health and Medical Research Council’s Australian Code of Practice for the Care and Use of Animals for Scientific Purposes (7th Edition 2004) and the Animal Care and Protection Act (2001).

### Perineural injection model

Micro-ultrasound guided perineural injection was performed in six to eight week old female CD1 wild-type mice.

The mice were anesthetized with 5% isoflurane in oxygen in an anesthetizing chamber and maintained with 1.5% isoflurane by nose cone for the duration of the procedure. The fur over the hind limb was clipped with an animal clipper. The mouse was turned to the lateral position and the hind limb was passed through an orifice in the bottom of a Petri dish sealed with a silicone membrane. The skin was prepared with 70% ethanol and allowed to dry. The Petri dish was filled with sterile phosphate buffered saline (PBS) to act as an ultrasound conduction medium for imaging, thus avoiding the use of ultrasound gel.

**Figure 1:**
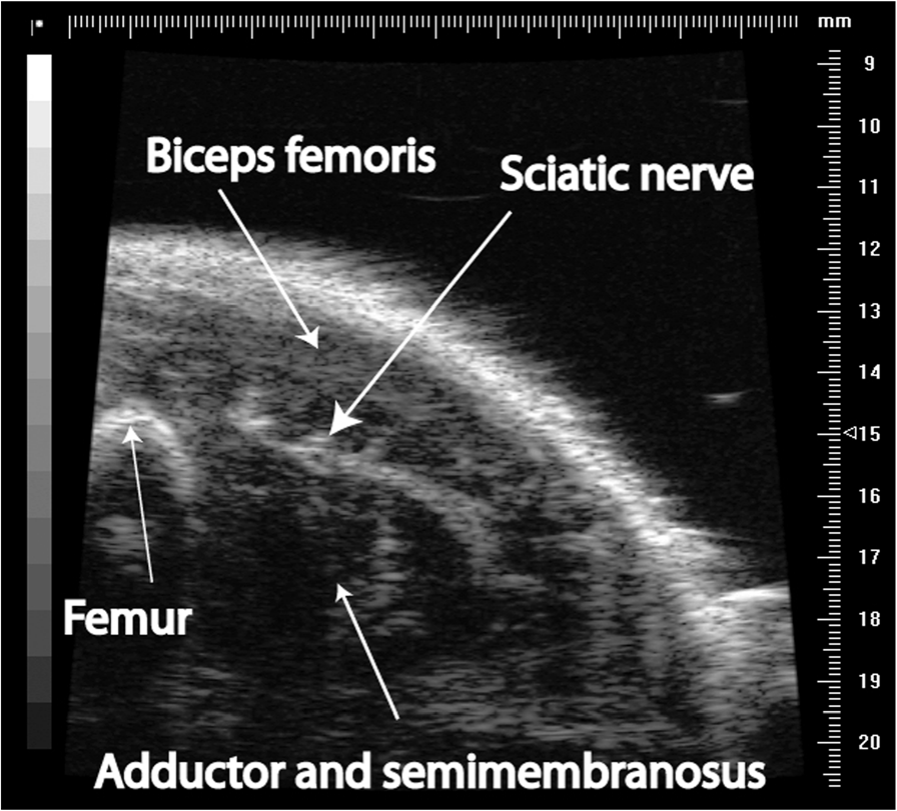
Ultrasound appearance of the sciatic nerve in the mouse.

A Vevo 770 (VisualSonics, Canada) micro-ultrasound machine with a 25 MHz probe was used to image a cross section of the sciatic nerve in the proximal hind limb. The leg was scanned proximally and distally to ensure consistent identification of the sciatic nerve. The sciatic nerve can be identified as a circular structure running in the fascial layer deep to the biceps femoris muscle at the mid-thigh (Figure 1).

**Figure 2:**
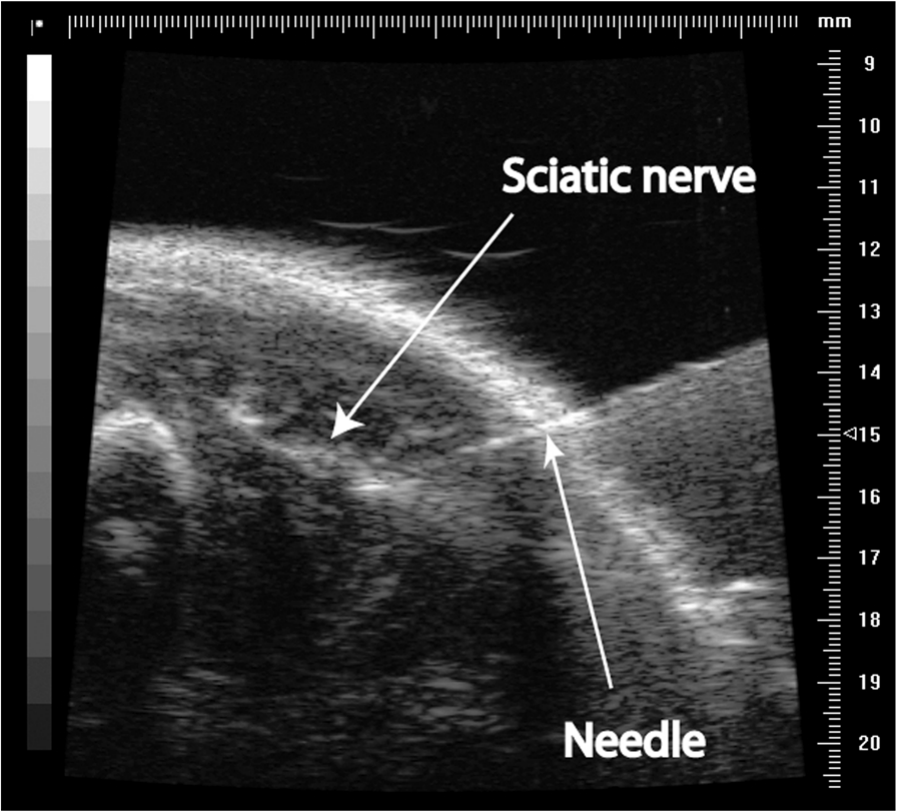
Needle insertion for perineural sciatic nerve injection.

**Figure 3:**
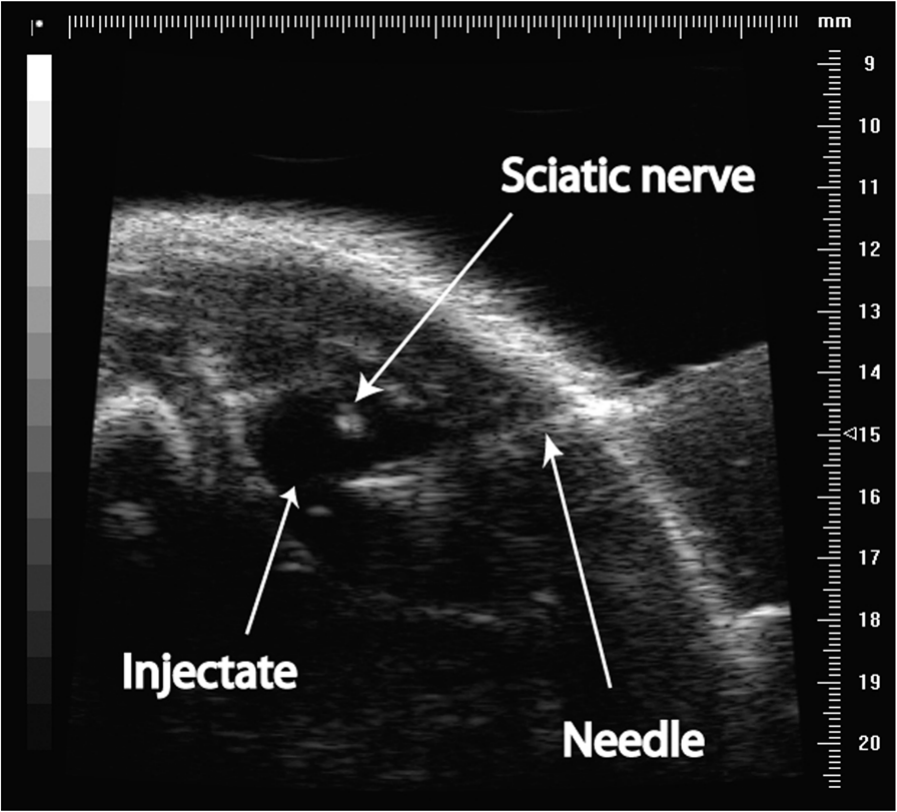
Injectate surrounding the sciatic nerve.

A 30 G needle was inserted into the thigh using an in-plane technique and the tip was positioned 0.5 mm away from the nerve, deep to the biceps femoris muscle (Figure 2). 0.1 ml of the test substance was injected at a single point. The injected fluid spread over the nerve (Figure 3). The preparation was mixed with 12.5 mcl/ml of 2 micrometre Fluoresbrite® Yellow Green Microspheres (Polysciences Inc, Warrington PA, USA). These inert, non-toxic polystyrene and latex microspheres are readily visible under ultraviolet light. They served as a visual marker of injection location during the dissection stage [16,17]. The investigators performing the injections were not blinded to the injected fluid.

The animals were kept for three days until euthanasia was performed. Three days was chosen because Schwann cell changes and macrophage migration begin to be visible two days after peripheral nerve injury [18]. After the initial myelin extrusion, Schwann cell division is maximal at three days [18]. The sciatic nerve was dissected with care to prevent crushing. The location of the injection around the sciatic nerve was confirmed by examination under ultraviolet light demonstrating fluorescence of the microspheres around the sciatic nerve. This area of nerve was sampled for histological assessment. The nerve was fixed and processed for histology. Semi-thin Toluidine blue transverse plastic sections were examined for nerve injury by two blinded neuropathologists. The nerves were assessed for axonal degeneration, myelin degeneration, intraneural and perineural inflammation on a qualitative scale (present, possible or absent).

### Propylene glycol concentration-neurotoxicity relationship

The first stage assessed the relationship between the concentration of PG injected and histological evidence of neurotoxicity in 42 animals.

Propylene glycol (Sigma Aldrich, Australia) was diluted with saline to make five solutions of 2.5%, 10%, 35%, or 70% (v/v) in addition to a saline control. For each concentration, eight animals had 0.1 ml of solution injected around one sciatic nerve. One additional experiment was performed with a saline control and 70% propylene glycol (n=9).

### Neurotoxicity assessment of gels

The second stage of the project was to perform perineural injection of three sterile gels: Aquasonic® 100 Ultrasound Transmission Gel (Parker Laboratories, USA); K-Y® Lubricating Jelly (Johnson & Johnson, USA); and PDI® Lubricating Jelly (Professional Disposables International, USA).

Each gel was tested in eight animals (n=24). Each animal had bilateral perineural injections with saline on one side and gel on the other. Sample size calculations were not performed because no data was available to provide an estimate of effect size. Descriptive statistics were used.

## Results

### Propylene glycol

Injections were performed with a saline placebo and varying concentrations of PG.

In the saline control group (n=9), perineural inflammation was present in five specimens (56%) (Table 1). One specimen (11%) showed no perineural inflammation and three (33%) showed subtle changes that were classified by the pathologists as “possible” perineural inflammation. Perineural inflammation was present with all injections of PG.

**Table 1:** Propylene glycol specimens showing confluent degenerative changes on histology

**PG concentration**	**Perineural inflammation**	**Confluent changes**		
		**Axon degeneration**	**Myelin degeneration**	**Intraneural inflammation**
	**n (%)**	**n (%)**	**n (%)**	**n (%)**
0% n=9	5 (56%) possible in 3 (33%)	0 (0%)	0 (0%)	0 (0%)
2.5% n=8	8 (100%)	0 (0%)	0 (0%)	0 (0%)
10% n=8	8 (100%)	0 (0%)	0 (0%)	0 (0%)
35% n=8	8 (100%)	0 (0%) *	0 (0%) *	2 (25%) *
70% n=9	9 (100%)	5 (56%)	5 (56%)	6 (66%)

PG: Propylene glycol.

* Two specimens showed patchy axonal and myelin degeneration with intraneural inflammation in smaller fascicles, rather than confluent changes.

In this model, a single isolated degenerating axon could be found in many specimens regardless of the injectate (Saline 8 of 9, 2.5% 3 of 8, 10% 4 of 8, 35% 6 of 8, 70% 7 of 9). As this was present across all concentrations and was not associated with intraneural inflammation, this pattern was considered to be normal for the model.

**Figure 4:**
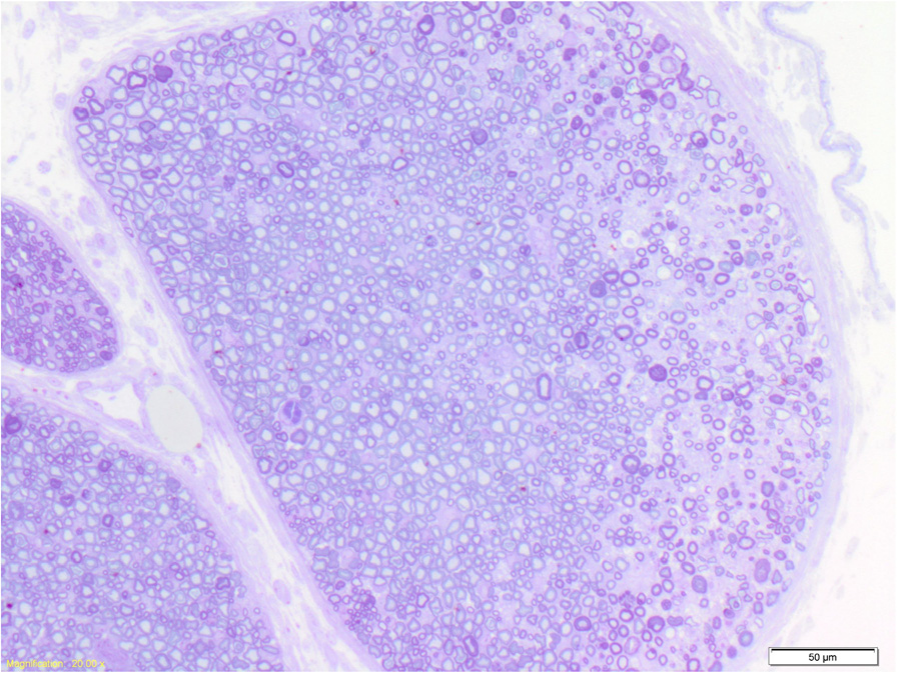
**Photomicrograph of mouse sciatic nerve (×200) three days after perineural injection of 70% propylene glycol.** The region to the left is normal. The region on the right demonstrates a peripheral area of axonal degeneration under the perineurium.

With the injection of 70% PG (n=9), five specimens (56%) demonstrated confluent areas of axonal degeneration and intraneural inflammation. At the level of injection, the sciatic nerve contained a number of fascicles. These confluent changes were restricted to a segment of a fascicle or several fascicles (Figure 4). The degenerative changes did not affect the entire nerve. The average depth of changes from the perineurium was 141 microns (SD 67 microns, range 40 – 205 microns) in the five specimens affected. This pattern of histological injury was only seen with the higher concentrations of propylene glycol (Table 1). Two specimens treated with 35% PG (n=8) showed patchy axonal and myelin degeneration with intraneural inflammation in smaller fascicles, rather than confluent changes. One of these specimens demonstrated capillary lumen occlusion and endothelial reaction indicative of focal vascular injury.

The analysis of the PG specimens demonstrated that PG injury is similar to glycerol, producing peripheral confluent areas of degeneration. These changes were assessed in the three gels studied.

### Gels

The histological findings for the gels are summarized in Table 2. None of the nerves in the Aquasonic® 100 or PDI® groups demonstrated confluent areas of axonal degeneration or intraneural inflammation.

**Table 2:** Histological findings for gels

	**n**	**Perineural inflammation**	**Confluent changes**		
			**Axonal degeneration**	**Intraneural inflammation**	**Myelin degeneration**
		**n (%)**	**n (%)**	**n (%)**	**n (%)**
Aquasonic® 100					
Gel	8	8 (100%)	0 (0%)	0 (0%)	0 (0%)
Saline	8	8 (100%)	0 (0%)	0 (0%)	0 (0%)
PDI®					
Gel	8	8 (100%)	0 (0%)	0 (0%)	0 (0%)
Saline	8	7 (82.5%)	0 (0%)	0 (0%)	0 (0%)
K-Y®					
Gel	8	8 (100%)	1 (13%)	1 (13%)	1 (13%)
Saline	8	5 (63%) Possible 2 (25%)	1 (13%)	1 (13%)	1 (13%)

One nerve treated with K-Y® Jelly showed confluent areas of axon loss affecting several fascicles. The contralateral nerve treated with saline did not show any changes. In another nerve treated with saline, one fascicle showed a peripheral wedge of injury with associated intraneural inflammation. The contralateral nerve treated with K-Y® did not show similar changes.

## Discussion

In this study, we used a murine model of perineural peripheral nerve injection to study the histological effects of PG and ultrasound gels. PG injection is associated with confluent areas of axon and myelin degeneration with intraneural inflammation. These changes were seen in five of eight specimens treated with 70% PG and, to a lesser degree, at 35% PG. This adds to existing knowledge that 80% and 100% PG causes complete or partial paralysis with diffuse degenerative changes of nerves from peripheral to central portions [9]. We did not find these changes at lower concentrations.

Perineural administration of 100% glycerol causes neurolysis from the peripheral areas of nerves, but these changes are not seen at 50% [8]. The histological appearance found with PG is similar to the neurolytic effect of glycerol [8]. The molecular mechanism of glycerol or propylene glycol neurolysis is unknown. Diffusion from the point of injection and direct chemical injury is likely [9]. Another potential cause for a wedge-shaped pattern is ischaemic injury caused by occlusion of vasa nervorum. The presence of an occluded capillary in one specimen supports the possibility of vascular injury and ischaemic neuropathy as a risk factor.

The concentrations of glycerol and PG present in the studied gels are below that likely to cause neurolysis after perineural injection [6]. This was confirmed for Aquasonic® 100 and PDI® lubricating gel, with no specimen demonstrating histological injury.

The results from K-Y® Jelly are more difficult to interpret. One nerve treated with K-Y® and a nerve treated with saline from a different animal demonstrated injury. No other specimen treated with saline showed similar changes suggesting that another process such as direct needle trauma or vascular injury may have been involved. These experiments were performed as a batch on one day and calls into question the changes found in the one K-Y® specimen.

Perineural inflammation was seen with all gels and propylene glycol. This is consistent with a previous study of ultrasound gel [14]. Pintaric and coworkers found that intrathecal injection of ultrasound gel in piglets increased protein levels in cerebrospinal fluid and caused infiltration of inflammatory cells into the meninges and spinal cord [15]. An inflammatory response occurs after perineural injection of ultrasound gel but there is no evidence that this is clinically significant.

We observed at least subtle perineural inflammation in the majority of saline placebo specimens. El-Dawlatly and coworkers did not observe perineural inflammation in their placebo group [14]. In their study, the posterior tibial nerves of dogs were exposed surgically without injection. This difference probably represents a difference between their canine and our murine models. Qualitatively, there was less perineural inflammation in the saline placebo group which is consistent with the findings of El-Dawlatly. Alternatively, perineural inflammation may occur from tissue dissection by injected fluid or the Fluoresbrite® microspheres.

The limitations of this study need to be considered prior to interpreting the results. This study only assessed histological injury at one time point (3 days) after perineural injection of gels. Testing at other time points or performing functional tests for the clinical features of nerve injury may have increased the sensitivity and clinical applicability. Not all specimens in the 70% propylene glycol group demonstrated confluent areas of axonal degeneration. While biological variability may explain some of the difference, variation in injection technique cannot be excluded. While an ultrasound guided injection approximates clinical practice, a surgical exposure may have yielded more consistent results. We also did not test intraneural injection which is likely to pose a greater risk for injury and is clinically relevant [8]. Finally, the pathologists analysing the specimens were blinded but there is a risk of bias because the investigators performing the injections were not.

In the clinical setting, the volume of gel carried by a needle will be small and will be diluted by the injected local anesthetic. Human nerves also have more abundant connective tissue than mouse nerves, [19] and therefore may have a greater chance of being protected from perineural alcohols. With these factors in mind, perineurial inflammation may occur clinically related to injected ultrasound gel, but it is unlikely to be of clinical significance. We are unaware of any clinical cases that have attributed nerve injury to ultrasound gel.

## Conclusions

The absence of histological evidence of neurolysis with perineural administration of Aquasonic® 100 and PDI® in this model argues against significant risk with passing the needle through the studied gels during regional anesthesia. Removing excess gel from the injection site may reduce perineural inflammation but this inflammatory response is of uncertain clinical significance.

## Competing interests

The authors declare that they have no competing interests.

## Authors’ contributions

DB conceived the study, participated in study design and conduct, and drafted the manuscript. NS participated in study design, conduct of laboratory work, and manuscript preparation. QL participated in study design, histological analysis of specimens, and manuscript preparation. TR participated in study design, histological analysis of specimens, and manuscript preparation. All authors read and approved the final manuscript.

## Pre-publication history

The pre-publication history for this paper can be accessed here:

http://www.biomedcentral.com/1471-2253/13/18/prepub
